# The sFlt-1/PlGF Index as an Auxiliary Tool in the Prediction of Adverse Perinatal Outcomes in Late-Onset Fetal Growth Restriction: A Systematic Review

**DOI:** 10.3390/biomedicines14061321

**Published:** 2026-06-10

**Authors:** Martyna Drzycimska, Magdalena Bednarek-Jędrzejek, Ewa Kwiatkowska, Sebastian Kwiatkowski

**Affiliations:** 1 Department of Obstetrics and Gynecology, Pomeranian Medical University, University Clinical Hospital No. 2, Aleja Powstańców Wielkopolskich 72, 70-111 Szczecin, Poland; m.bednarekjedrzejek@gmail.com (M.B.-J.); kwiatkowskiseba@gmail.com (S.K.); 2 Department of Nephrology, Transplantology and Internal Medicine, Pomeranian Medical University, University Clinical Hospital No. 2, Aleja Powstańców Wielkopolskich 72, 70-111 Szczecin, Poland; ewakwiat@gmail.com

**Keywords:** fetal growth restriction, sFlt-1/PlGF ratio, angiogenesis markers, adverse perinatal outcomes

## Abstract

**Background/Objectives:** This study evaluates the prognostic utility of placental insufficiency biomarkers in late-onset fetal growth restriction occurring after 32 weeks of gestation. These markers were assessed both independently and in conjunction with ultrasound and Doppler indices to predict adverse perinatal outcomes. Given the clinical integration of maternal angiogenic factor assessment, it is imperative to determine whether these markers—specifically the sFlt-1/PlGF ratio—can effectively mitigate fetal and neonatal morbidity and mortality. **Methods:** This review is registered with PROSPERO and encompasses the literature from the last decade. The present analysis incorporated prospective observational cohort studies. Four comprehensive databases were queried, resulting in the evaluation of fifty-one relevant records. Thirty-six full-text articles were assessed for eligibility, resulting in the inclusion of six relevant manuscripts. The total sample size was 14.499 patients. Detection rates for growth restricted fetuses demonstrated significant variability, ranging from 15% to 88.5%. **Conclusions:** The clinical utility of angiogenic biomarkers for the prediction of fetal growth restriction remains unresolved; moreover, the empirical evidence synthesized within this review exhibits a significant degree of heterogeneity.

## 1. Introduction

Currently, screening strategies focus on antenatal ultrasound to detect small fetuses. However, this ultrasound-based strategy ignores the underlying pathological processes in the placenta. Among the small-for-gestational-age (SGA) neonates, some have suffered from fetal growth restriction (FGR), while the rest are healthy and constitutionally small due to their demographic factors. Significantly, critical confounding variables in the clinical management of late-onset fetal growth restriction (FGR) include maternal obesity, advanced maternal age, anthropometric parameters, parity, and substance misuse, specifically involving smoking cigarettes and cocaine use.

SGA fetuses are defined as those having gestational weights below the tenth percentile with normal Doppler flow spectra. In contrast, fetal growth restriction, i.e., the restriction of genetically predetermined growth potential due to placental dysfunction, is a major cause of neonatal morbidity and mortality. During the prenatal period, SGA fetuses are detected at a rate of approximately 5% to 8% of all late-phase pregnancies [1]. SGA may be associated with an increased risk of adverse perinatal outcomes (APOs). There is a general consensus that, especially for late-onset small fetuses, there should be a clinical distinction between high-risk FGR, in most cases associated with placental insufficiency and worse perinatal outcomes, and low-risk SGA, usually associated with outcomes similar to those in fetuses of the appropriate gestational age (AGA) [2].

Globally, almost 20 million infants are born with low birth weight—many of whom are growth-restricted [3]. FGR affects approximately 10% of pregnancies and is one of the leading causes of preterm birth and neonatal death, as well as various neonatal short- and long-term morbidities. Given that the fetus fails to achieve its full growth potential, there is an increased predisposition toward obesity, type 2 diabetes, and cardiovascular disease in later life. Furthermore, these individuals are subject to a heightened risk of neurological disorders, encompassing cognitive impairments, learning disabilities, and behavioral or motor dysfunctions. Consequently, systematic longitudinal monitoring of developmental progress during the early postnatal years is imperative.

The pathophysiology of late FGR differs from that of early FGR. Consequently, alterations in UA Doppler and venous districts are rare and, most importantly, fail to identify the vast majority of late-FGR cases or to predict adverse outcomes in these fetuses. The differentiating features of early- and late-onset FGR are shown in Table 1.

Proper placental development requires synchronization of a number of angiogenic markers. Failure to maintain the establishment of a low-pressure vascular interface between the mother and fetus leads to placental insufficiency, which is causally linked to intrauterine growth restriction. Specifically, the roles played by two angiogenic factors—soluble fms-like tyrosine kinase 1 (sFlt-1), which is an antiangiogenic protein, and placental growth factor (PlGF), which is a proangiogenic protein—have been extensively researched in the recent literature.

Of note, the field of placental pathology is rapidly changing with relevant data generated regarding the ability to predict preeclampsia (PE), as well as intrauterine growth restriction, preterm delivery, and stillbirth [4].

FGR identification by routine ultrasound or symphysis–fundal height is suboptimal, leading to delayed diagnosis. It has been shown that the addition of markers based on the identification of placental dysfunction, such as the mean uterine artery pulsatility index (mUtA PI) and/or umbilical artery pulsatility index (UA PI) and the sFlt-1/PlGF ratio, improves the detection of early FGR cases as well as PE [5,6,7].

In view of this, the time has come to reveal the role of these factors in FGR diagnosed beyond 32 + 0 weeks gestation as well as to anticipate adverse outcomes.

Although some reports suggest that incorporation of the sFlt-1/PlGF ratio might be helpful in the management of and differentiation between SGA and FGR, the lack of interventional trial data precludes the recommendation of these tests as an adjunct to ultrasound imaging [8,9,10,11,12,13]. There are few reports of such an association with late-onset FGR. Data from reliable studies are sparse, hence the need for this systematic review.

The objective of this study was to analyze the sFlt-1/PlGF ratio alone or in combination with ultrasound examination for prediction of late-onset FGR and adverse perinatal outcomes.

## 2. Methods and Results

### 2.1. Search Strategy

This systematic review was prospectively registered in the PROSPERO database (CRD420251077900) on 20 June 2025, prior to data extraction, and adhered to the PRISMA (Preferred Reporting Items for Systematic Reviews and Meta-Analyses) guidelines. A comprehensive literature search was conducted across PubMed, Scopus, Embase, and Web of Science, covering the decade from 2015 to the present. The search strategy employed the following keywords: “late-onset fetal growth restriction” AND “sFlt-1/PlGF ratio” OR “angiogenesis factors” AND “adverse perinatal outcomes”.

The initial search identified 51 records, from which 15 duplicates were removed. After screening titles and abstracts, 36 full-text articles were evaluated for eligibility, ultimately yielding six relevant manuscripts for inclusion. All included studies were prospective observational cohorts; review articles were excluded from the final selection. To ensure literature saturation, reference lists of the included manuscripts were manually screened. Data extraction and quality assessment were performed independently by two authors. The study population consisted of singleton pregnancies complicated by late-onset fetal growth restriction occurring after 32 weeks’ gestation. In accordance with the Delphi consensus, this was defined as an estimated fetal weight (EFW) below the 3rd percentile or between the 3rd and 10th percentiles when accompanied by feto-maternal Doppler abnormalities.

Exclusion criteria encompassed multiple gestations, early-onset FGR or small-for-gestational-age (SGA) fetuses diagnosed prior to 32 + 0 weeks (defined as EFW below the 10th percentile without Doppler abnormalities), and studies investigating angiogenic markers other than sFlt-1 and PlGF.

### 2.2. Data Extraction

**Figure 1:**
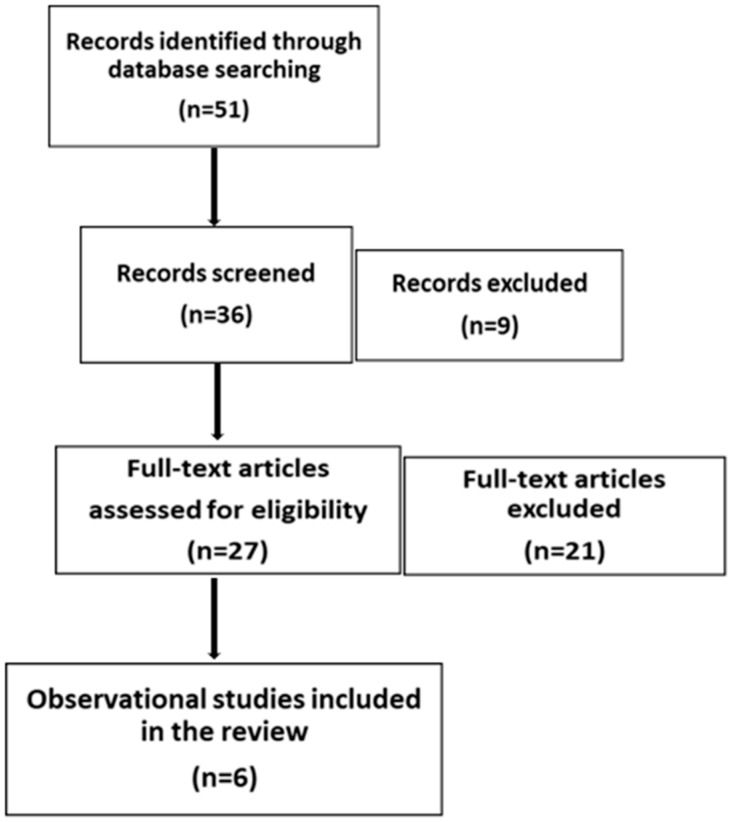
PRISMA flow chart of the systematic review.

Data from the eligible studies were extracted and evaluated independently by two reviewers. Information retrieved from the full-text articles encompassed secondary pregnancy complications, cohort sizes, and primary conclusions regarding the prediction of adverse perinatal outcomes relative to the implemented screening protocols: estimated fetal weight, the sFlt-1/PlGF ratio (applied individually or in combination), and Doppler parameters. The study selection process is illustrated in the PRISMA flow diagram (Figure 1).

### 2.3. Quality Assessment

The methodological quality of the included studies was evaluated using the Quality Assessment of Diagnostic Accuracy Studies-2 (QUADAS-2) instrument. Risk of bias was appraised within four primary domains: (1) patient selection, (2) index test, (3) reference standard, and (4) flow and timing. These findings are illustrated in Figure S1. Risk of bias in each study [2,9,14,15,16,17,18] (Supplementary Material).

### 2.4. Data Synthesis

We summed up the data in appropriate tables and figures and used mean values to represent the records. Furthermore, the data were divided into multiple categories, of which the most relevant were those containing information regarding the false-positive ratios (FPR) and detection rate (DR) values and their importance in making the diagnosis of late-onset FGR cases and predicting adverse outcomes. All the results are presented to two decimal places.

## 3. Results

Two studies out of six indicated that angiogenic markers provided only minimal to no discernible enhancement in prognostic accuracy for the delivery of low-birth-weight infants below the 10th and 3rd percentiles. The detection rates for a model incorporating maternal characteristics and estimated fetal weight ranged from 23% to 90% for fetuses below the 10th percentile and from 23% to 88% for fetuses below the 3rd percentile. Upon the integration of angiogenic markers, predictive performance was observed to slightly increase (by four percentage points) for fetuses with an estimated fetal weight below the 10th percentile. In contrast, fetuses falling below the third percentile exhibited a marginal increase of only two percentage points. 

Subsequent investigations have corroborated the clinical utility of incorporating angiogenic biomarker assays into predictive models for preterm delivery, specifically concerning gestations concluding prior to 37 weeks and instances of late-onset fetal growth restriction with EFW below the 3rd percentile. The most robust predictor of adverse pregnancy outcomes was identified as a multi-parametric profile comprising fetal weight below the 3rd percentile, increased uterine artery pulsatility indices, and an inverted cerebroplacental ratio, integrated with an sFlt-1/PlGF ratio exceeding the 95th percentile.

## 4. Discussion

Our review includes papers that, in the last 10 years, have investigated the utility of the sFlt-1/PlGF ratio in predicting adverse perinatal outcomes in late-onset FGR cases. Scientific sources on this topic are poor. Nonetheless, the data are encouraging, and we should not stop at current patterns of daily practice. This paper provides evidence on the predictive value of angiogenic factors, especially when used together as a ratio for adverse perinatal outcomes among late-onset small fetuses accompanied by ultrasound and Doppler parameters.

SGA fetuses generally receive routine prenatal care with full-term delivery and have been considered to be constitutionally small babies with good perinatal outcomes. However, it has long been acknowledged that SGA neonates are at increased risk of perinatal mortality and morbidity and have a higher risk of developing disabilities in childhood [13]. An optimal screening strategy for the prediction of SGA, and mainly those with FGR, is highly desirable. One of the most feared complications by obstetricians and perinatologists is stillbirth. It is a devastating and often preventable adverse pregnancy outcome. Monitoring levels and trends of this setback is essential to further progress in reducing pregnancy loss. Improving the health of mothers, newborns and children has long been the focus of large-scale global public health efforts. In order to differentiate between SGA and FGR or to identify the latter among the SGA in cases in which the fetal size is below the 10th percentile, additional biophysical parameters are required. Many methods have been proposed for this purpose, such as evaluation of fetal growth velocity, use of customized growth charts, Doppler velocimetric evaluation of placental and fetal circulations, and use of biomarkers [8].

The latest nomenclature distinguishes between SGA and FGR. The latter is sometimes referred to in the literature as the severe form of SGA. However, it is important to note that some studies use these terms interchangeably, which puts a crucial bias into the analysis and is confusing. Therefore, in our review, we only analyzed small fetuses with EFW <3rd percentile or between the 3rd and 10th centiles with abnormalities in Doppler flow spectra, that is, fetuses with growth restriction. The first approaches regarding the use of angiogenesis markers as predictors of poor perinatal outcomes were put forward by Fadigas et al. [14]. The investigators assessed the clinical utility of individual placental markers, specifically PlGF and sFlt-1, rather than their ratio, at 35–37 weeks’ gestation for predicting small-for-gestational-age neonates in the absence of preeclampsia. Their screening methodology incorporated maternal characteristics—such as age and ethnicity—alongside estimated fetal weight calculated from head circumference (HC), abdominal circumference (AC), and femur length (FL). Notably, the authors stratified small fetuses into three categories based on birth weight percentiles (<10th, <5th, and <3rd), designating the latter as growth-restricted fetuses.

It was observed that the sFlt-1 marker did not provide essential independent prediction of SGA in the absence of PE, in addition to combined testing by maternal factors and fetal biometry at 35–37 weeks’ gestation. On the other hand, the addition of PlGF alone only slightly improved the performance of screening for small fetuses (Table 2). 

Similar conclusions were reached by Ciobanu et al. [15]. The results spoke of a subtle improvement in the anticipation of adverse perinatal outcomes by the use of biomarkers of impaired placentation separately (similar to their predecessors). In this case, fetal weight was divided into two degrees of severity of SGA: <10th and <3rd percentiles for gestational age at delivery. 

According to the authors, fetal assessment at 36 weeks of gestation, including a combination of maternal variables, EFW, and biophysical and biochemical markers of a compromised placenta, could predict roughly 90% of SGA newborns born within 2 weeks of evaluation with a positive-screen rate of approximately 20% and 90% of SGA newborns born at any time after assessment with a positive-screen rate of as high as 30% (Table 2). To simplify, these fetuses are about one-third more likely to be small for gestational age. This indicates the need for further diagnostic testing to distinguish growth-restricted fetuses, which are the main cause of many adverse neonatal outcomes requiring hospitalization in the neonatal intensive care unit (NICU) and neonatal mortality. It is known that up to 40% and even up to 50% of fetuses classified as SGA will have FGR, which poses a diagnostic challenge.

Novel insights were provided by Gaccioli et al., who utilized the Delphi consensus criteria to define fetal growth restriction [9]. The study population was stratified into two gestational cohorts at 28 and 36 weeks, with assessments of the sFlt-1/PlGF ratio and estimated fetal weight (EFW). Results demonstrated that the positive likelihood ratios at both gestational ages were superior to those derived from conventional definitions of suspected FGR based exclusively on ultrasound assessment (Table 2). At 36 weeks’ gestation, the primary outcome was the delivery of a small-for-gestational-age neonate with associated complications. In this group, 102 participants (3%) tested positive for both markers, in contrast to 1% in the 28-week cohort. The positive predictive value (PPV) was 21.60% (14.50–30.80%), and the screen-positive cohort accounted for more than one-third of women who delivered a complicated SGA infant.

Finally, by combining fetal biometry and the calculation of the sFlt-1/PlGF ratio, the PPV was over 20% at 28 and 36 weeks of gestation, and the false-positive rates (FPRs) were 0.90% at 28 weeks and 2% at 36 weeks. This approach enabled the identification of women at high absolute risk of adverse pregnancy outcomes, with a very low FPR in the healthy population. 

The work of Visan et al. yielded results turning the scales in favor of angiogenic markers, specifically the sFlt-1/PlGF ratio, similarly to Gaccioli et al. [16]. The FGR definition adopted by the researchers was the one provided by the American College of Obstetricians and Gynecologists (ACOG): estimated fetal weight less than the 10th percentile for gestational age. This study supported the researchers’ initial hypothesis that combining the sFlt-1/PlGF ratio with ultrasound biometry could enhance the sensitivity of screening tests for identifying small fetuses beyond 37 weeks’ gestation. When ultrasound biometry alone was used to estimate fetal weight below the 10th percentile, the sensitivity of the method was significantly lower at 44.40% and the specificity was 89% with a false-positive rate (FPR) of 10%. When ultrasound was combined with an sFlt-1/PlGF ratio (cut-off value ≥38), sensitivity nearly doubled, reaching 84.21% with a specificity of 84.31%, which is relevant because the FPR was the same (Table 2).

More importantly, this threshold remained unchanged when all cases of small fetuses were considered, including those complicated by preeclampsia in ongoing pregnancy, or when only cases of FGR without associated preeclampsia were incorporated. It is vital because, when screening specifically for late-onset FGR, we can use the same cut-off value of 38 as for PE. Nevertheless, further empirical investigation involving a more extensive sample size is required to formulate definitive conclusions regarding the utility of the indicator within this specific context.

It can be safely concluded that PE is associated with an almost threefold greater risk of fetal growth restriction and that these conditions often occur concomitantly, which correlates with higher sFlt-1/PlGF ratios [17]. Therefore, the authors excluded patients who had developed preeclampsia so as not to impact the results of the study.

In 2023, Hurtado et al. targeted the establishment of a predictive model incorporating placental biomarkers and their potential to predict adverse perinatal outcomes in low-birth-weight fetuses in late pregnancy (Table 2) [18]. SGA was defined as EFW between the 3rd and 10th percentiles without abnormalities in fetal and maternal Doppler studies, and FGR was defined as EFW below the 3rd percentile or between the 3rd and 10th percentiles concurrent with abnormalities in fetal and maternal Doppler studies. The findings demonstrated that when comparing both models, the model without the sFlt-1/PlGF ratio was significantly less effective than the model with the sFlt-1/PlGF ratio (59.30% vs. 85.30%, respectively) in predicting preterm delivery before 37 weeks of gestation and adverse perinatal outcomes. In cases where delivery is anticipated before 35 weeks of pregnancy, the use of the UtA PI measurement model has proven beneficial when the sFlt-1/PlGF ratio is not available. The AUC was 0.89 for the use of Doppler imaging instead of the sFlt-1/PlGF ratio (AUC 0.93; 95% CI). The detection rates of adverse perinatal outcomes for each model are provided in Table 2. 

Ultimately, at least one adverse perinatal outcome was observed in 43 (37.4%) pregnancies. APO may be defined as any of the following: stillbirth, intrapartum cesarean section due to non-reassuring CTG, neonatal death, NICU admission for longer than 48 h, respiratory distress syndrome (RDS), bronchopulmonary dysplasia (BPD), neonatal sepsis, retinopathy of prematurity (stages III–IV), periventricular leukomalacia, Apgar score < 7 at 5 min or umbilical artery cord pH < 7.

The latest study by Youssef et al. showed that the sFlt-1/PlGF ratio alone had a low predictive value for adverse perinatal outcomes, but when combined with ultrasound measurement (EFW), its predictive effectiveness was similar to that of EFW in combination with Doppler parameters, 39.80% vs. 81.30% vs. 86.80%, respectively [2]. The model combining the sFlt-1/PlGF ratio with EFW and Doppler indices achieved the highest APO detection rate, reaching 88.50%. It should be noted that the highest FPR was recorded in the model including all three components.

Notably, the SGA and FGR criteria were the same as in previous studies. Last but not least, in settings of limited access to Doppler assessment, the sFlt-1/PlGF index can be combined with EFW measurements with a high detection rate and a lower FPR compared to combining them with Doppler indices [2]. Estimated fetal weight below the third percentile remains the strongest predictor of poor outcome among small fetuses [2,18]. It should also be remembered that conversely, when it is not possible to perform placental insufficiency calculations, EFW below the 3rd percentile combined with UtA-PI over the 95th percentile and a calculated cerebro-placental ratio (CPR) <5th percentile is also a sensitive method for identifying growth-restricted fetuses with a high detection rate.

Screening and interventions based on a combination of a suspected small fetus on ultrasound and blood tests indicating placental insufficiency might have a real chance of demonstrating clinical effectiveness in reducing adverse pregnancy outcomes associated with fetal growth restriction, preferentially after 32 weeks of gestation. Such research should be a targeted area for future studies.

We suggest implementing a protocol integrating ultrasonography (with Doppler indices) with the analysis of biochemical markers, specifically the sFlt-1/PlGF ratio. This methodology facilitates the identification of pregnancies at high risk for late-onset fetal growth restriction, potentially mitigating the incidence of such complications. A proposed threshold value of ≥40 for the sFlt-1/PlGF ratio is suggested [19]. This cut-off demonstrates significant utility in predicting preterm delivery and severe perinatal complications, thereby supporting clinical decision-making. However, this index should not be utilized as a singular diagnostic criterion but rather interpreted alongside maternal medical history and clinical presentation.

## 5. Strengths and Limitations/Risk of Bias Assessment

The following limitations have been established for the scope of this study: (1) the absence of a meta-analysis, (2) the lack of discussion of publication bias, (3) the small number of included studies despite a ten-year search period, and (4) the fact that two included studies had “some concerns” on QUADAS-2 that were not addressed in the interpretation.

Different fetal and neonatal growth charts with various cut-off values are applied around the world to define FGR and SGA. Thus, it is challenging to clearly define fetal growth restriction. It is usually defined based on the statistical deviation of fetal size from the population pattern, with a threshold at the 10th, 5th, or 3rd percentile, hence the discrepancies. This is heavily misleading. However, the threshold is more useful for SGA, based on actual weight at birth. In the absence of a gold standard for defining FGR based on underlying pathophysiology, SGA/severe SGA is used as an inadequate proxy that represents a heterogeneous group of both constitutionally small and intrauterine-growth-restricted fetuses.

The heterogeneity could also be caused by varying study designs and using different assays. The studies included in this review encouragingly used the same immunoassays for PlGF and sFlt-1, such as Cobas e411 Roche Diagnostics [9,14,15,18] and Cobas 6000 [16], with one exception, where ELISA kits were used (R&D Systems Europe Ltd. [2]). Unfortunately, standardization of immunoassays is difficult and rather unfeasible nowadays. Most of the works cited include large groups, which is a strong point of the review. However, as mentioned earlier, the papers covered in the analysis include cases of mixed small-for-gestational0age/fetal growth restriction, which is perplexing. The index test standard (domain D2/D3) results were not interpreted without knowledge of the results of the reference standard. Lack of blinding could potentially lead to bias, as the person who was interpreting the index test was influenced. Unfortunately, the examiner was already aware of the diagnosis. The main limitation was also that the results of fetal biometry were made available to the obstetricians of the patients. Further, they took specific actions for further monitoring of cases of suspected fetal growth restriction and, consequently, the performance of screening, particularly for those delivering within a short period of time (2 weeks of assessment).

## 6. Conclusions

Evidence from six prospective cohort studies suggests that integrating the sFlt-1/PlGF ratio with estimated fetal weight significantly enhances the detection rate of adverse perinatal outcomes in late-onset fetal growth restriction. Specifically, detection rates increased from approximately 44% utilizing ultrasonography in isolation to 81–85% when combined, maintaining false-positive rates between 2% and 10%. Nevertheless, substantial heterogeneity in threshold values and outcome definitions, coupled with a lack of interventional trial data, currently prevents the endorsement of this approach for routine clinical application. The question of their clinical usefulness for monitoring and birth planning in FGR pregnancies diagnosed beyond 32 weeks’ gestation still remains unclear. Consequently, large-scale prospective validation studies employing standardized protocols are warranted.

We intended to emphasize how relevant it is to seek additional and accessible tools that may increase the level of identification of these cases and distinguish them from constitutionally small fetuses and anticipate adverse perinatal outcomes as well. These promising tools are markers of angiogenesis, the sFlt-1/PlGF ratio, fully accepted by the scientific field.

## Figures and Tables

**Table 1:** Main clinical characteristics of early- and late-onset fetal growth restriction.

Characteristic/Feature	Early-Onset Fetal Growth Restriction	Late-Onset Fetal Growth Restriction
Incidence	20–30%	70–80%
The key clinical challenge	Management	Detection
Gestational age at the time of onset	Before 32 weeks	32 weeks and beyond
Perinatal mortality	High	Low
Placental abnormalities	Anomalies at the implantation stage and within spiral arteries, insufficient blood supply to the placenta	Primarily altered diffusion, fewer typical changes in the placenta

**Table 2:** Characteristics of analyzed studies.

No.	Author, Year, Type of Study	Number of Cases (n)	Additional Pregnancy Complications	EFW and/or sFlt-1/PlGF Ratio or Each Separately and/or Doppler Parameters Main Findings for Prediction of Adverse Perinatal Outcome (APO)	Conclusion
**1.**	Fadigas et al., 2015 [14], prospective screening	n = 3701 - reference group, n = 158 - SGA group at 35–37 GA	- Chronic hypertension - preexisting diabetes mellitus	1. Combined screening by maternal factors and EFW predicted 90%, 92% and 94% of SGA neonates with birth weight < 10th, <5th and <3rd percentiles delivering <2 weeks following assessment, respectively. 2. Combined screening by maternal factors, EFW and serum PlGF predicted 88%, 96% and 94% of SGA neonates with birth weight < 10th and <3rd percentiles delivering <2 weeks of assessment, respectively.	PlGF alone marginally improves the performance screening of SGA fetuses.
**2.**	Gaccioli et al., 2018 [9], prospective cohort study	n = 3981 women were analyzed for 28 GA and n = 3747 women were analyzed for 36 GA	- Chronic hypertension - renal disease	1. At both 28 and 36 weeks of gestational age, the positive predictive value was around twice as high for combined ultrasonic and biochemical screening compared with the best-performing purely ultrasonic method, 21.30% vs. 3.80% and 21.60% vs. 7.50%, respectively. 2. At 36 GA, PlGF was equally predictive of late fetal growth restriction as the sFLT1/PlGF ratio. However, sFLT1 was only slightly weaker or similar as a predictor compared to the sFLT1/PlGF ratio at 36 GA.	A combination of ultrasonic suspicion of a small fetus and a blood test indicating placental dysfunction was strongly predictive of clinically important adverse pregnancy outcomes.
**3.**	Ciobanu et al., 2019 [15], comparison of two datasets	n = 19,209 - training dataset - including n = 1012, SGA fetuses - validation dataset - n = 1012, SGA fetuses at 35–37 GA	- Chronic hypertension - diabetes mellitus type 1 and 2	1. Screening by maternal factors and EFW predicted 75% and 85% of SGA neonates with birth weight < 10th and <3rd percentiles delivering within 2 weeks of assessment. 2. Detection rate equal 90% of SGA < 10th percentile delivering within 2 weeks- the necessary screen-positive rate would be 67% in screening by maternal factors, 23% by maternal factors and EFW and 21% by the maternal factors, EFW and UtA PI + MCA PI + PlGF; the respective values for SGA < 3rd percentile were 63%, 18%, and 15%, respectively.	Angiogenesis markers only marginally improve the predictive performance for delivery of SGA fetuses.
**4.**	Visan et al., 2019 [16], prospective case–control study	n = 74, including n = 37 - FGR arm, n = 37 - control arm at 33 GA	- Chronic hypertension - diabetes mellitus	1. When ultrasound (US) biometry and maternal risk factors were used to estimate EFW < 10 percentiles, the sensitivity was 44.4% with a specificity of 89%. 2. When the aforementioned variables were associated with the sFlt1/PIGF ratio, for a cut-off of 38, the sensitivity was 84.21%, and the specificity was 84.31%.	Supplementary use of the sFlt-1/PlGF ratio, in addition to maternal factors and US biometrics, enhanced the sensitivity for detecting late FGR.
**5.**	Hurtado et al., 2023 [18], prospective cohort study	n = 115, including n = 55, SGA n = 60, FGR at 32 + 0 and 36 + 6 GA	9 cases - PE 4 cases - APS	1. The multivariate model including sFlt-1/PlGF (and fetal Doppler assessment * + GA in weeks) showed a better predictive performance for APO and delivery before 37 WG than the multivariate model without sFlt-1/PlGF ratio. 48.80% vs. 34.90% and 85.20% vs. 59.30%, respectively.	sFlt-1/PlGF index is a good predictor of APO at the time of late-onset FGR/SGA diagnosis.
**6.**	Youssef et al., 2025 [2], prospective cohort study	n = 602, including n = 189, SGA n = 413, FGR at 34 GA	91 (15.1%) developed preeclampsia, including 85 cases of FGR	1. EFW < 3rd centile + sFlt-1/PlGF ratio > 95th centile Detection rate: 81.30%. 2. EFW < 3rd centile + UtA PI > 95th centile + CPR < 5th centile Detection rate: 86.80%. 3. EFW < 3rd centile + UtA PI > 95th centile + CPR < 5th centile + sFlt-1/PlGF ratio > 95th centile Detection rate: 88.50%.	Combining sFlt-1/PlGF ratio with EFW and Doppler indices achieved the highest detection rate for adverse perinatal outcomes.

* UA PI > 95th percentile, UA AEDF or CPR < 5th percentile; UtA PI > 95th percentile, EFW percentile.

## Data Availability

No new data were created or analyzed in this study. Data sharing is not applicable to this article.

## Supplementary Materials

The following supporting information can be downloaded at: https://www.mdpi.com/article/10.3390/biomedicines14061321/s1, Figure S1: Risk of bias in each study.

## Abbreviations

The following abbreviations are used in this manuscript:

EFW	Estimated Fetal Weight
UA	Umbilical Artery Pulsatility Index
UtA PI	Uterine Artery Pulsatility Index
MCA	Middle Cerebral Artery Pulsatility Index
CPR	Cerebro-Placental Ratio
GA	Gestational Age
WG	Weeks’ Gestation
APS	Antiphospholipid Syndrome
PE	Preeclampsia
SGA	Small for Gestational Age
FGR	Fetal Growth Restriction
sFLT-1/PlGF	soluble fms-like tyrosine kinase 1/placental growth factor ratio
APO	Adverse Perinatal Outcomes
AGA	Appropriate for Gestational age
FPR	False-Positive Ratio
DR	Detection Rates
HC	Head Circumference
AC	Abdominal Circumference
FL	Femur Length
NICU	Neonatal Intensive Care Unit
PPV	Positive Predictive Value
FPR	False-Positive Rate
AEDF	Absent End-Diastolic Flow
CTG	Cardiotocography
AUC	Area Under the Curve
CI	Confidential Interval
