# Supplementary material for: The sFlt-1/PlGF Index as an Auxiliary Tool in the Prediction of Adverse Perinatal Outcomes in Late-Onset Fetal Growth Restriction: A Systematic Review
Source: Biomedicines. 2026 Jun 10;14(6):1321. doi: 10.3390/biomedicines14061321 (PMC13296736; doi:10.3390/biomedicines14061321)
Supplement: Supplementary file 1 [file biomedicines-14-01321-s001.zip › biomedicines-4299152-supplementary.pdf]

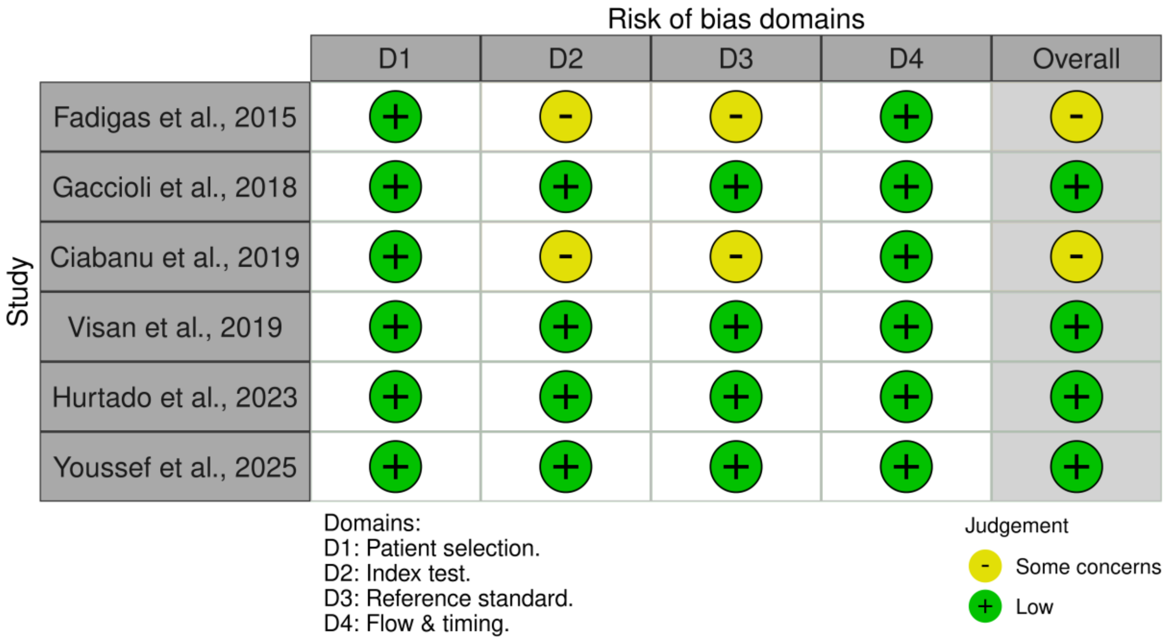

Figure S1. Risk of bias in each study
